# Factors affecting decisions to use antibiotic-sparing treatment approaches in women with uncomplicated urinary tract infections: a scoping review protocol

**DOI:** 10.11124/JBIES-24-00159

**Published:** 2025-04-02

**Authors:** Stefania Di Gangi, Stefan Neuner-Jehle, Robin Baumann, Andreas Plate

**Affiliations:** 1 Institute of Primary Care, University of Zurich and University Hospital Zurich, Zurich, Switzerland

**Keywords:** antibiotic-sparing treatment, barriers, facilitators, urinary tract infection

## Abstract

**Objective:**

This scoping review will explore the evidence on factors influencing the decision to use antibiotic-sparing treatments in women with uncomplicated urinary tract infections.

**Introduction:**

Overuse and misuse of antibiotics are the main drivers of antimicrobial resistance. Antibiotic-sparing treatments, such as symptomatic treatment with analgesics and delayed prescriptions, have considerable potential to reduce antibiotic consumption, but the majority of patients still receive antibiotics without delay. The reasons for the poor implementation of these alternative approaches are unknown. A better understanding of the factors influencing treatment decisions is needed.

**Inclusion criteria:**

This review will consider the experiences and attitudes of health care professionals and women in outpatient settings in high-income countries, regarding the use of antibiotic-sparing treatments for the diagnosis or suspected diagnosis of uncomplicated urinary tract infections (ie, healthy women aged 18–64 years who are not pregnant, immunosuppressed, and have no functional or structural urinary tract abnormalities).

**Methods:**

This review will follow the JBI methodology for scoping reviews. MEDLINE (Ovid), Embase, and the Cochrane Library will be searched to identify peer-reviewed articles: original research (quantitative or qualitative studies, experimental, or observational), reviews, case reports, and case series. Gray literature will also be searched for. Sources in any language from 2000 to 2024 will be included. Three reviewers will screen the sources and extract data using a tool developed by the reviewers. The analysis will use counts and descriptive qualitative content analysis. The results will be presented in visual, tabular, and narrative formats.

**Review registration:**

Open Science Framework osf.io/t8y5e/

## Introduction

Antimicrobial resistance (AMR) is a major global health threat and was associated with nearly 5 million deaths in 2019.^1^ A report commissioned by the UK government predicted that AMR could cause up to 10 million deaths per year by 2050.^2^ Overuse and misuse of antibiotics are the main drivers of rising AMR rates. Up to 80% of all antibiotics are prescribed in primary care^3^ and urinary tract infections (UTIs) are one of the most common reasons for prescribing antibiotics, second only to respiratory tract infections.^4^,^5^

UTIs are associated with a substantial burden for health care systems.^1^ One in 3 women will experience a UTI episode by the age of 24, and about one out of 2 women will experience at least one UTI episode during her lifetime.^6^ Due to the frequent prescription of antibiotics, patients with UTIs are confronted with the various drawbacks of antibiotic use, such as adverse drug reactions (eg, gastrointestinal complaints or allergic reactions) or secondary infections (eg, *Clostridium difficile*, candida). Moreover, antibiotic use is strongly associated with AMR,^2^,^3^ which has public health implications.

Clinically, UTIs are classified as uncomplicated or complicated.^7^ Uncomplicated UTIs (uUTI), the most common scanario, usually refer to acute infections in premenopausal and non-pregnant women without known urological abnormalities or comorbidities.^8^ Complicated UTIs are usually associated with being male, pregnancy, indwelling catheters, urinary tract abnormalities, immunosuppression, or extensive exposure to antibiotics.^7^ Uncomplicated UTIs may not always require rapid treatment, as symptoms may resolve spontaneously.^9^

Most interventions in uUTI, such as educating physicians about AMR, clinical practice guidelines, and educational sessions on prescription habits, are effective in improving the choice of appropriate antibiotic treatment and increasing awareness of this public health concern.^10^ However, as both appropriate and inappropriate use of antibiotics accelerate the development of AMR, new approaches without antibiotics are of great interest. Alternative and complementary therapies using vaccines and medical plants show potential in the treatment of uUTI.^11^ Another option to reduce antibiotic consumption, and, therefore, the burden at patient and public health levels, is antibiotic-sparing treatment (AST), such as symptomatic treatment with analgesics and/or intentionally delayed antibiotic prescriptions.^7^,^12^ In a recent meta-analysis, AST approaches reduced antibiotic consumption by up to 63%.^12^ Symptomatic treatment and the option of delayed antibiotic use are recommended as valid treatment options for women with uUTI in national and international guidelines.^13^-^15^ Women are generally willing to delay antibiotic treatment for uUTI,^16^ but the available evidence suggests that antibiotics are still prescribed immediately in most consultations for women with uUTI.^17^-^19^ The reasons for this remain unknown.

Despite the reduction in antibiotic use, current evidence suggests that the use of AST is also associated with drawbacks,^12^ such as prolonged symptom burden or increased risk of developing pyelonephritis. It is unknown whether these effects, or other unknown factors, influence the decision to use AST. It is also unclear which factors facilitate the decision to use AST. Prior research suggests that to identify these factors, both patient and health care provider attitudes to AST should be considered, as treatment for UTIs should be part of a shared decision-making process.^20^ Given the high incidence of uUTI in women, better implementation of AST could have great potential to reduce antibiotic consumption, and consequently, the individual, economic, and public health burden of uUTI.

Therefore, the objective of the proposed scoping review is to explore the extent of the literature from developed countries on the factors (eg, barriers, facilitators) that influence the decision to use AST in women with uUTI. A preliminary search of PubMed and *JBI Evidence Synthesis* was conducted and no current or in-progress scoping reviews on the topic were identified. A scoping review is the most appropriate approach to assess and understand the extent of knowledge in an emerging field; to identify the types of evidence in a field; or to identify, map, report, or discuss the key characteristics or factors related to a concept.^21^ This scoping review will identify and analyze knowledge gaps to inform research and policy on AST. The review may serve as a foundation for systematic reviews to inform future research on AST, taking into account the barriers and facilitators identified, and to validate their role in clinical practice.

## Review questions

What are the factors (eg, facilitators, barriers) affecting decisions to use AST regimens in women with uUTI?

- What factors could be identified as facilitators of the decision to use AST?
- What factors could be identified as barriers to the decision to use AST?
- Are there other factors that could affect the decision to use AST?
- What are the specific factors, facilitators, or barriers related to the provider’s profession (eg, general practitioner, pharmacist, other health professional)?
- What are the knowledge gaps regarding attitudes toward AST?

## Inclusion criteria

### Participants

This review will consider studies that include health care professionals and women who have used, directly or indirectly experienced, or expressed attitudes toward AST or antibiotic prescribing for the diagnosis of acute uUTI or suspected acute uUTI. *Acute* is defined as the new onset of symptoms (within days) as opposed to symptoms over weeks. It should be noted that there are some discrepancies between the definitions of uUTI in the literature.^22^ Therefore, a specific definition is not proposed. Studies on uUTI will be included if the criteria for defining the condition are consistent with the general understanding of an uncomplicated UTI, namely, an infection in a woman, aged 18–64 years, with no underlying health problems and no specific anatomic or functional abnormalities in the urinary tract.^15^

Based on the available evidence and guidelines on UTI,^15^ this review will exclude sources with a primary or exclusive focus on UTI in children or adolescents (aged under 18 years), older women (aged 64 years and over), men, pregnant women, relevant immunosuppression, functional or anatomical abnormalities of the urogenital system, catheter-associated UTI, and hospital-associated UTI. All of these are considered complicated UTI.

### Concept

This review will include research that reports on factors affecting the decision to use AST, such as experiences, attitudes, and beliefs that may act as facilitators or barriers. Experiences include any feedback, opinion, or perception about AST from the general population, patients, health care professionals (eg, physicians, pharmacists). *Barriers* and *facilitators* are defined as factors that may have a direct or indirect impact on the decision to use AST, either hindering or enabling it. For example, if a physician is concerned about the higher risk of pyelonephritis with AST compared to standard care, this will be considered a barrier from a health care provider’s perspective. Another barrier may be patients’ expectations that they will be given antibiotics at the first consultation for a UTI. On the other hand, a facilitator for AST could be training for physicians to improve their knowledge and practice of AST or patient knowledge about AST.

For the purposes of this review, AST is defined as any of the following:

- Symptomatic treatments recommended by a health care provider, which include all non-antimicrobial treatments such as painkillers, herbal remedies, or increased fluid intake.
- Delayed prescription of antibiotics, where patients are recommended to delay the use of antibiotics.^12^,^20^ All approaches to delayed prescribing will be considered, in line with prior research:^23^ patient-led strategy (patient fills the prescription but is advised to wait until there is no spontaneous improvement), forward dating of the prescription, returning to the practice to collect the prescription, or re-consultations (in the practice or by telephone) to decide on antibiotic treatment.
- Combination of symptomatic treatment with delayed prescription approaches.

### Context

This review will consider all evidence from outpatient settings, such as primary care, general practice, family medicine practices, emergency rooms, or pharmacies. Only high-income countries will be considered, as defined by the World Bank Country and Lending Groups classification in 2024.^24^ Community pharmacies will be considered as they provide self-care advice but also dispense antibiotics for uUTI in urgent situations and may influence patient behavior through their recommendations.^25^

Evidence from inpatient settings and long-term care, including nursing homes, will be excluded. Low- and middle-income countries will be excluded because of their different health systems, infection control, regulations, resources, and the use of antibiotics for self-medication, given their availability over-the-counter in many countries.

### Types of sources

This scoping review will consider both experimental and quasi-experimental study designs, including randomized controlled trials, non-randomized controlled trials, before-and-after studies, and interrupted time series studies. In addition, analytical observational studies, including prospective and retrospective cohort studies, case-control studies, and analytical cross-sectional studies, will be considered for inclusion. This review will also consider descriptive observational study designs, including case series, individual case reports, and descriptive cross-sectional studies. Guidelines published in peer-reviewed journals or studies that review guidelines will also be considered. Qualitative and mixed methods studies will also be considered. In addition, reviews of any type that meet the inclusion criteria will be considered. Text and opinion papers; gray literature such as conference proceedings, theses, dissertations, government documents, policy documents, and clinical practice guidelines; and books will also be considered for inclusion.

## Methods

The proposed review will be conducted in accordance with the JBI methodology for scoping reviews^26^ and reported in line with the Preferred Reporting Items for Systematic Reviews and Meta-Analyses extension for Scoping Reviews (PRISMA-ScR).^27^

### Search strategy

The search strategy will aim to locate published primary studies and reviews. An initial limited search of MEDLINE (Ovid), Embase, and the Cochrane Library (CDSR and Trials) database was undertaken to identify articles on the topic. The text words contained in the titles and abstracts of relevant articles, and the index terms used to describe the articles, were used to develop a full search strategy for MEDLINE (Ovid; see Appendix I). The search strategy, including all identified keywords and index terms, will be adapted for each included information source. The search strategy was developed in collaboration with an experienced librarian, who will carry out the entire search. The search strategy will be based on 4 concepts: (i) UTIs, (ii) antibiotics, (iii) antibiotic-sparing treatments, and (iv) facilitators and barriers. The reference lists of all sources meeting the inclusion criteria will be manually back-searched for additional studies.

Articles in any language will be included. Where necessary, studies will be translated into English using DeepL software (DeepL, Cologne, Germany). Exclusion of studies due to lack of translation will be documented in the review. Articles from 2000 to the present will be included, as the concept of deliberately delaying prescriptions or using symptomatic treatments to spare antibiotics had not emerged before 2000.^23^ Therefore, literature prior to 2000 will be excluded. The databases to be searched will include MEDLINE (Ovid), Embase, and the Cochrane Library. Sources of unpublished studies and gray literature to be searched will include government or organization websites, clinical guidelines, ProQuest Dissertations and Theses Global, and Google search (limited to the first 10 pages to maximize relevance).

### Source of evidence selection

Following the search, all identified records will be collated and uploaded into EndNote v.21.2 (Clarivate Analytics, PA, USA) and duplicates removed. Following a pilot test, and due to the heterogeneity of what may be considered a facilitator or barrier, the titles and abstracts will be screened by 3 reviewers, who will independently decide whether to include or exclude each source and then discuss it together. After familiarizing themselves with the topic through the title and abstract screening process, 2 or 3 reviewers will assess the full text of potentially relevant sources in detail against the inclusion criteria. Reasons for the exclusion of full-text sources that do not meet the inclusion criteria will be recorded and reported in the scoping review. Any disagreements that arise between the reviewers at each stage of the selection process will be resolved by discussion or with an additional reviewer. The results of the search and the study inclusion process will be fully reported in the final scoping review and presented in a PRISMA flow chart.^28^

### Data extraction

Data will be extracted by 2 or more independent reviewers using a data extraction tool developed by the reviewers. The extracted data will include specific details about the participants, concept, context, study methods, and key findings relevant to the review questions. A draft extraction tool has been developed, adapting the template provided in the JBI methodology for scoping reviews^26^ (see Appendix II). The data extraction tool will be pilot-tested using 10 sources that will include a mix of original research. At least 2 reviewers will independently pilot-test the data extraction tool, and necessary modifications to the tool will be made following discussion. To allow for emergent data throughout the data extraction process, the data extraction tool will be modified and revised as necessary. Modifications will be detailed in the scoping review and any disagreements that arise between the reviewers will be resolved through discussion or with an additional reviewer. If appropriate, authors of papers will be contacted up to 2 times to request missing or additional data. Critical appraisal of individual sources of evidence is generally not required for scoping reviews and will not be undertaken.

### Data analysis and presentation

Quantitative and qualitative data will be analyzed according to the methodological guidance for scoping reviews.^29^ Characteristics of sources of evidence will be reported in tabular format. Common frequencies regarding the number of evidence sources that used a particular method (eg, RCTs, surveys) and the location, country, or context where the evidence source was conducted will be reported. As appropriate, a world heat map will be created, with the size of the circle indicating how many evidence sources were conducted in that country. For the main research question, basic qualitative content analysis will be carried out using an inductive approach. The results will be presented in a chart to demonstrate the frequency of the themes and the categories describing barriers and facilitators affecting decisions to use AST, according to the corresponding provider (physician or pharmacist) and to patients or the general population. Other appropriate forms of visual presentation may be used, depending on the results. It is important to note that perceptions of barriers and facilitators may differ between health care providers and patients. The barriers and facilitators identified in this review will be primarily categorized according to their assessment in the included publications and evaluated from a clinical perspective. Further qualitative studies may be needed to determine the true perceived categorization in a specific health care setting among physicians and patients. A narrative summary will accompany the presentation of results. Specific information about potential conflicts of interests will be summarized in a supplementary table.

## Acknowledgments

University Library of Zurich, Switzerland, for assistance in developing the search strategy.

## Author contributions

All authors have contributed to the conceptualization, methods, and writing of the scoping review protocol and have read and agreed to the submitted version.

## Appendix I: Search strategy

### MEDLINE (Ovid)

Search conducted: September 30, 2024

**Table:**

Search	Query	Records retrieved
1	Urinary Tract Infections/ or exp Bacteriuria/ or exp Pyuria/ or Cystitis/	56,399
2	(bacilluria or bacteruria or bacteriuria or cystiti* or pyuria* or urocystiti* or ((urinary-tract or urine or urologic*) adj6 infection*) or (bladder adj3 (inflammation* or infection*)) or ((purulent or pus) adj3 urine)).ti,ab.	73,592
3	1 or 2	94,258
4	Anti-Bacterial Agents/ or Anti-Infective Agents/ or exp Anti-Infective Agents, Urinary/	502,489
5	(antibiotic* or anti-biotic* or antimicrobial* or anti-microbial* or antibacterial* or anti-bacterial* or antiinfective* or anti-infective*).ti,ab.	692,845
6	4 or 5	882,952
7	exp Antimicrobial Stewardship/ or exp Time-to-Treatment/	14,374
8	(door-to-treatment-time* or onset-to-treatment-time* or symptom-to-treatment-time* or time-to-therap* or time-to-treatment* or ((antibiotic* or anti-biotic* or antimicrobial* or anti-microbial*) adj3 stewardship*) or ((antibiotic* or anti-biotic* or antimicrobial* or anti-microbial* or therap* or treatment* or prescribing* or prescription*) adj3 (delay* or postpone*)) or ((antibiotic* or anti-biotic* or antimicrobial* or anti-microbial* or therap* or treatment*) adj3 alternative*) or (back-up* adj3 (antibiotic* or anti-biotic* or antimicrobial* or anti-microbial* or prescribing* or prescription*)) or ((reduc* or decreas* or cutback* or diminution* or sparing*) adj3 (antibiotic* or anti-biotic* or antimicrobial* or anti-microbial*)) or ((non-antibiotic* or non-anti-biotic* or non-antimicrobial* or non-anti-microbial* or antibiotic-sparing* or anti-biotic-sparing* or antimicrobial-sparing* or anti-microbial-sparing* or stand-by* or standby* or self-care* or selfcare* or symptomatic*) adj3 (strateg* or therap* or treatment*)) or (culture adj3 (antibiotic* or anti-biotic* or antimicrobial* or anti-microbial*))).ti,ab.	207,270
9	7 or 8	216,427
10	Decision Making/ or exp Decision Making, Shared/ or “Attitude of Health Personnel”/ or “Patient Acceptance of Health Care”/ or Patient Compliance/ or exp Patient Participation/ or exp Patient Satisfaction/ or Education/ or Health Education/ or Patient Education as Topic/ or Knowledge/ or Attitude to Health/ or exp Health Knowledge, Attitudes, Practice/ or risk factors/ or Pyelonephritis/ or exp Pyonephrosis/	1,740,154
11	(attitude* or accepta* or choice* or choicemaking or decision* or decisionmaking or role* or experience* or practice* or compliance* or engagement* or expectation* or feeling* or involv* or inexperience* or intention* or knowledge* or motivation* or participation* or preference* or satisfaction* or willingness* or understanding* or understood or paradigma* or education* or consider* or risk or risks or benefit* or beneficial* or facilitator* or favour* or favor* or oppos* or perspective* or advice* or advis* or hazard* or recover* or factor* or variable* or pyelonephros* or pyonephros* or uroseps* or pyelonephriti* or (taken adj3 seriously) or (change adj3 (behavior* or behaviour* or practice* or prescribing* or prescription*)) or ((kidney or renal) adj3 infection*) or ((kidney or renal) adj3 (abscess* or carbuncle*)) or (infected adj3 hydronephrosis) or ((pyelonephrotic or pyonephrotic) adj3 kidney) or ((urinary or uro) adj3 seps*) or ((purulent or pus) adj3 urine) or (bladder adj3 inflammation*)).ti,ab.	17,190,081
12	10 or 11	17,525,232
13	3 and 6 and 9 and 12	2211
14	13 not (animals not humans).sh.	2164
15	14 not ((exp child/ or exp infant/ or exp adolescent/) not exp adult/)	1955
16	limit 15 to yr = “2000 - 2024”	1821

## Appendix II: Data extraction instrument

**Table:**

Evidence source details and characteristics		
Paper ID		
Citation details		
Year		
Journal/place		
First author (last name only)		
Title		
Country of origin		
Study design (eg, review, qualitative, quantitative, mixed methods)		
Type of evidence source (eg, peer-reviewed, gray)		
Aims/purpose/objective of source		
Study population		
Sample size		
Description of the setting primary care/pharma/other		
Details extracted from source of evidence (in relation to the concept of the scoping review)		
Definition and description of the antibiotic-sparing treatment used (eg, symptomatic treatment, delayed prescription, combination)		
Key findings related to the research question	Experiences	
	Facilitators	
	Barriers	
	Other	
